# Treatment of Poor Sperm Quality and Erectile Dysfunction With Oral Pentoxifylline: A Systematic Review

**DOI:** 10.3389/fphar.2021.789787

**Published:** 2022-01-12

**Authors:** Yi Lu, Hao Su, Jianzhong Zhang, Yutao Wang, Hongjun Li

**Affiliations:** ^1^ Department of Urology, Peking Union Medical College Hospital, Peking Union Medical College, Chinese Academy of Medical Sciences, Beijing, China; ^2^ Department of Urology, Beijing Friendship Hospital, Capital Medical University, Beijing, China; ^3^ Department of Urology, China Medical University, The First Hospital of China Medical University, Shenyang, China

**Keywords:** pentoxifylline, male infertility, sperm, erectile dysfunction, oligoasthenoteratozoospermia

## Abstract

**Background:** Pentoxifylline (PTX) is a member of methylxanthine chemicals and a type of non-selective phosphodiesterase-5 inhibitors, which has been used in male infertility treatment to improve sperm quality and erectile dysfunction (ED) treatment. Mutually tight associations existed between ED and male infertility. Using PTX might kill two birds with one stone by improving sperm quality and erectile function in infertile men with ED.

**Methods:** PubMed, Cochrane Library, EMBASE, and Web of Science were searched by October 2021. Based on available evidence from observational studies and randomized-controlled trials (RCTs), we conducted a systematic review to summarize the efficacy and safety of PTX in treating ED and male infertility. The protocol of the article was registered and updated in PROSPERO (CRD42021291396).

**Results:** From 202 records, eight studies (7 RCTs) evaluating the role of PTX in ED and three studies (2 RCTs) assessing the efficacy of PTX in male infertility were included in the systematic review. Three studies (100.00%) and two studies (100.00%) reported the beneficial role of PTX in improving sperm progressive motility and normal sperm morphology rate, respectively. In contrast, only one study (33.33%) indicated the favorable role of PTX in enhancing sperm concentration. As for ED, three (60.00%) studies supported the treatment role of PTX alone in ED, and two studies (66.67%) favored the combination use of PTX and selective PDE5Is compared with selective PDE5Is alone. Safety analysis showed that PTX was a well-tolerated drug in ED and male infertility treatment.

**Conclusion:** Given the association between ED and male infertility and satisfying findings from available evidence, PTX administration for the simultaneous treatment of poor sperm quality and mild ED in infertile men will highly enhance the treatment compliance. However, the finding should be treated carefully until validated by further studies.

## Introduction

Male infertility, a prevalent condition affecting millions of couples, is not only a medical issue but also a huge social problem. Research on this issue is becoming much more crucial in the context of population aging (Fisher and Hammarberg, 2012; Inhorn and Patrizio, 2015). At present, drugs, surgical interventions, and artificial reproductive technology are frequently used treatment in male infertility, while empirical drug intervention with a long history is still the most commonly used method for the treatment of oligoasthenoteratozoospermia (OAT) (de Kretser, 1997; Bisht et al., 2017). Furthermore, the application of modern reproductive technologies is often assisted by feasible drugs for increasing pregnancy and success rate (Henkel and Schill, 2003). Owing to conflicting results, poor study design, or statistical power, no potent drugs can achieve absolutely reliable efficacy in male infertility (Li, 2014; Minhas et al., 2021). However, patients still commonly prefer medication first, striving for natural conception, and non-invasive drug interventions are often empirically applied prior to more advanced technologies (Buckett and Sierra, 2019). Since the 1970s, various drugs have been used in the treatment of male infertility, including gonadotrophins, anti-estrogens, testosterone, anti-oxidation, and trace elements (Li, 2014; Carson and Kallen, 2021). Although the pathological and physiological basis of idiopathic male infertility has not been elucidated clearly and no strong evidence supporting drug treatment in male infertility is available (Minhas et al., 2021), we should not stop our discovery in proving drug effectiveness, and more rigorous investigations should be advocated and encouraged.

For a long time, methylxanthines have attracted huge research interest due to their wide use in assisted reproduction technology (ART). Pentoxifylline (PTX) is one of the methylxanthines and is now most frequently used in the treatment of sperm *in vitro* in ART (Mahaldashtian et al., 2021). Because of its concrete mechanisms and favorable role in microcirculation, oral PTX has been introduced into the treatment of male infertility since 1979 (Heite, 1979). After that, several laboratory and clinical studies were conducted to explore mechanisms on sperm and evaluate the efficacy and safety of its use in the human body *in vivo*. According to the review by Lotti et al., the prevalence of ED in infertile men ranged from 6.7 to 61.6% worldwide, causing serious harm to male health (Lotti and Maggi, 2018). Additionally, it was reported that the occurrence of erectile dysfunction (ED) was up to 57.8% in Chinese infertile men, while 60.38% of ED patients were presented with mild ED (Yang et al., 2018). As a member of nonselective phosphodiesterase inhibitors, PTX can also mildly increase the blood flow in the penis. With these underlying mechanisms, PTX may improve both sperm quality and erectile function in infertile men with ED at the same time. According to the PICOS strategy, we assessed the role of PTX in the treatment of male infertility and erectile dysfunction through a systematic review.

## Methods

### Evidence Acquisition

We made detailed inclusive criteria according to the well-established report guidelines before searching the literature (Stroup et al., 2000; Knobloch et al., 2011). In October 2021, all available evidence in PubMed, Web of Science, Cochrane Library, and EMBASE was systematically searched. Both observational studies and randomized controlled trials (RCTs) were included. References and citations of related articles were also searched carefully. The search process was performed independently by three authors. The keywords for the search were “pentoxifylline,” “erectile dysfunction,” and “male infertility” (details are shown in Supplementary Table S1.) The protocol of the article was registered and updated in PROSPERO (CRD42021291396).

### Inclusion and Exclusion Criteria

Studies that met the following criteria were included: 1) population: men with ED or male infertility; 2) interventions: PTX alone or combined with other drugs for assistance; 3) comparators: patients not using PTX or using placebos; 4) outcomes: including at least the evaluation on erectile function, or semen parameters or pregnancy outcomes; and 5) study design: observational studies or RCTs. Studies that failed to meet the inclusive criteria were excluded.

### Data Collection

Three authors screened retrieved literature independently. Information including the first author, publication year, study design, regions, demographic features, grouping information, and outcomes were recorded from included studies. Missing or unclear information was collected by contacting the article authors. When there is no reply from authors, corresponding information will be considered as “not available.”

### Risk of Bias Assessment

The Cochrane Collaboration’s tool for assessing the risk of bias in the trial and Newcastle–Ottawa Scale for cohort studies was used to evaluate the RoB by three authors independently (Peterson et al., 2011; Savović et al., 2014). Disagreements in the assessment were solved by discussion among the three authors and communication with article authors.

### Evidence Synthesis

Given limited evidence and the expected heterogeneity of studies from study design, patients, and interventions (duration, dosage, and types) and diverse criteria for OAT, it was difficult to quantitatively assess available evidence by conducting a meta-analysis. We cannot get any persuasive results with enough statistical power. Instead, we performed the descriptive analysis of patients, interventions, the study design, and main findings. According to the recommendation from *Cochrane Handbook for Systematic Reviews of Interventions*, we counted the number of favorable studies and compared it with the number of unfavorable studies (Cumpston et al., 2019). In each study, results were evaluated according to whether the authors found statistically significant evidence regarding the efficacy or safety of PTX. Review questions were determined by the balance of favorable versus unfavorable studies. Two-tailed *p* < 0.05 was considered statistically significant.

## Results

### Literature Selection

**FIGURE 1:**
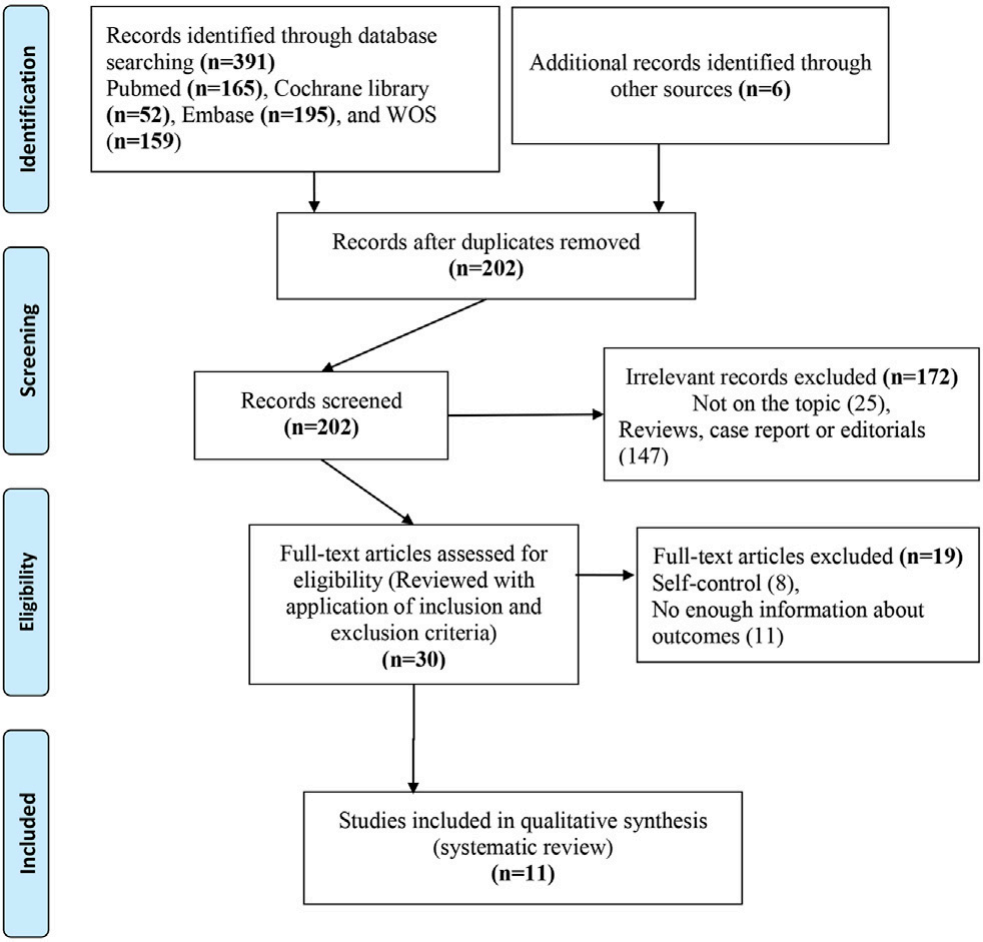
PRISMA flowchart of the data search.

Under the established search strategy, we found 202 non-repeated records. After the screening and eligibility evaluation, three studies demonstrating the role of PTX oral administration in male infertility and its impact on sperm parameters and eight studies on males with ED were included in the narrative analysis (Micic et al., 1988; Korenman and Viosca, 1993; Georgitis and Merenich, 1995; Knoll et al., 1996; Peşkircioğlu et al., 1996; Ozdal et al., 2008; Safarinejad, 2011; Zahran et al., 2012; Kumar et al., 2015; Nasimi Doost Azgomi et al., 2018; Law et al., 2020) (shown in Figure 1).

### Characteristics of Included Studies

**FIGURE 2:**
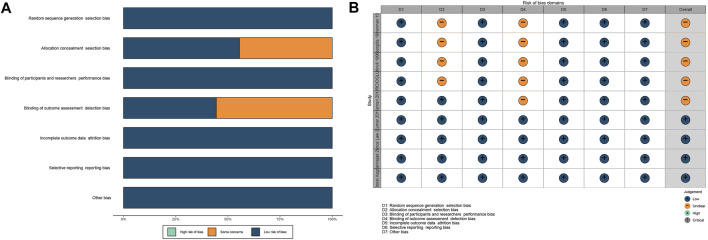
RoB of RCTs. **(A)**: percentage; **(B)**: traffic light. Abbreviations: RoB: risk of bias; RCT: randomized controlled trial.

With strict inclusive and exclusive criteria, limited evidence was recorded and analyzed. Details about the effect of PTX on sperm concentration, motility, and morphology are summarized in Tables 1–3, respectively. Some of them are PTX alone in male infertility, and some are in combination with other drugs. Additionally, there were eight studies focusing on the role of PTX in ED treatment, among which PTX dosage is consistent (all 400 mg, tid). Two prospective cohort studies and 9 RCTs were included in the analysis. RoB analysis indicated that all of them had an overall fair or high quality (shown in Figure 2 and Supplementary Table S2).

**TABLE 1:** The effect of oral PTX on sperm concentration.

Study	Study design	Intervention and control	Dosage (mg/d)	Duration of treatment (months)	No. of patients	Sperm concentration (×10^6^/ml)		Criteria for oligozoospermia (×10^6^/ml)	Conclusion
						Before treatment	After treatment		
Micic et al. (1988)	Prospective study	PTX and untreated	3 × 400	3	90	21.4 ± 29.4	20.1 ± 12.1	≤20	PTX oral administration cannot improve sperm concentration
Safarinejad (2011)	RCTs	PTX and placebo	2 × 400	4	254	16.2 ± 3.4	26.4 ± 4.6^a^	<20	PTX oral administration can improve sperm concentration in men with idiopathic OAT
Nasimi Doost Azgomi et al. (2018)	RCTs	PTX and *Withania somnifera*	800	3	100	56.68 ± 28.92	55.68 ± 27.52	<20	PTX oral administration cannot improve sperm concentration in idiopathic male infertility

a Statistically significant between pre-treatment and post-treatment.

Abbreviations: PTX: pentoxifylline; OAT: oligoasthenoteratozoospermia.

**TABLE 2:** The effect of oral PTX on sperm motility.

Study	Study design	Intervention and control	Dosage (mg/d)	Duration of treatment (months)	No. of patients	Sperm motility progressive (%)		Criteria for asthenozoospermia (%)	Conclusion
						Before treatment	After treatment		
Micic et al. (1988)	Prospective study	PTX and untreated	3 × 400	3	90	23.5 ± 8.6	31.2 ± 10.7^a^	<40	PTX oral administration can improve sperm motility
Safarinejad (2011)	RCTs	PTX and placebo	2 × 400	4	254	26.4 ± 2.4	35.8 ± 4.2^a^	<50	PTX oral administration can improve sperm motility in men with idiopathic OAT
Nasimi Doost Azgomi et al. (2018)	RCTs	PTX and *Withania somnifera*	800	3	100	17.71 ± 8.63	22.31 ± 11.58^a^	<50	PTX oral administration can improve sperm motility in idiopathic male infertility

a Statistically significant between pre-treatment and post-treatment.

Abbreviations: PTX: pentoxifylline; OAT: oligoasthenoteratozoospermia.

**TABLE 3:** The effect of oral PTX on sperm morphology.

Study	Study design	Intervention and control	Dosage (mg/d)	Duration of treatment (months)	No. of patients	Normal morphology (%)		Criteria for teratozoospermia (%)	Conclusion
						Before treatment	After treatment		
Safarinejad (2011)	RCTs	PTX and placebo	2 × 400	4	254	17.4 ± 4.2	25.4 ± 4.3^a^	<30	PTX oral administration can improve normal sperm morphology rate in men with idiopathic OAT
Nasimi Doost Azgomi et al. (2018)	RCTs	PTX and *Withania somnifera*	800	3	100	14.68 ± 6.34	16.63 ± 6.28^a^	<30	PTX oral administration can improve normal morphology rate in idiopathic male infertility

a Statistically significant between pre-treatment and post-treatment.

Abbreviations: PTX: pentoxifylline; OAT: oligoasthenoteratozoospermia.

### Effect of Pentoxifylline on Sperm Concentration

Conflicting results were found in three studies (Micic et al., 1988; Safarinejad, 2011; Nasimi Doost Azgomi et al., 2018), only one (33.33%) RCT reported the advantageous role of PTX in sperm concentration (Safarinejad, 2011). The authors conducted a well-designed study with a feasible sample size and a follow-up of 4 months, reporting results about sperm parameters changes, hormone changes, and pregnancy outcomes. By comparing with placebos, they found that using oral PTX (400 mg, bid) could significantly improve the sperm concentration in men. However, another two studies (66.67%) (cohort study conducted by Micic et al. and the RCT performed by Nasimi Doost Azgomi et al.) indicated no improvement in sperm concentration after the oral administration PTX. These studies have many differences in study design and diverse dosages. Few of them mentioned sperm volume or total sperm numbers, which can better reflect the effect of PTX on spermatogenesis.

### Effect of Pentoxifylline on Sperm Motility

Sperm concentration and sperm morphology are relatively stable and hard to modify after sperm formation. In contrast, sperm motility is more changeable through drug therapy. Results on this among the included studies are relatively consistent (Micic et al., 1988; Safarinejad, 2011; Nasimi Doost Azgomi et al., 2018). As shown in Table 2, all of them (100.00%) reported a significant improvement after PTX oral administration, while different criteria for sperm progressive motility might influence the judgment of the findings.

### Effect of Pentoxifylline on Sperm Morphology

Only two RCTs were included in terms of the sperm morphology (Safarinejad, 2011; Nasimi Doost Azgomi et al., 2018). With the same total dosage, the authors undertook different dosage frequencies: one is 400 mg, bid. for 1 day (Safarinejad, 2011) and another one is 800 mg/day (Nasimi Doost Azgomi et al., 2018). However, they both (100.00%) found that PTX oral administration can improve normal morphology rate in idiopathic male infertility.

### Pentoxifylline in the Treatment of ED

Five of them reported the single-use of PTX in erectile function (Georgitis and Merenich, 1995; Knoll et al., 1996; Korenman and Viosca, 1993; Peşkircioğlu et al., 1996; Zahran et al., 2012), three (60.00%) indicated improvement in erectile function through objective assessment after using PTX (Knoll et al., 1996; Korenman and Viosca, 1993; Peşkircioğlu et al., 1996) (Table 4). Korenman et al. conducted a small RCT to evaluate the role of PTX in treating vascular ED. By measuring patients’ reports, partners’ verification, and penile-brachial pressure index, they found PTX can be a well-tolerated alternative treatment for mild-to-moderate vascular ED (Korenman and Viosca, 1993). Although Georgitis et al. concluded that pentoxifylline is not an effective treatment for diabetic ED through results from objectively measured percent rigidity, they indeed reported eight patients (among 34 individuals) were benefited from PTX administration (Georgitis and Merenich, 1995). Subsequently, Zahran and his colleagues investigated the efficacy of PTX in treating ED patients after receiving a T-shunt procedure and reported that PTX had no significant effect on the recovery of erectile function after severe ischemic injury (Zahran et al., 2012). It is not difficult to understand that, without good control of blood sugar level or surgical repair, the efficacy of pharmacological treatment of diabetic, surgery, or complex vascular injuries caused-ED is certainly limited. Three studies are about the combination use of sildenafil or tadalafil and PTX in ED (Ozdal et al., 2008; Kumar et al., 2015; Law et al., 2020), two (66.67%) of them supported the combination use of PTX and selective PDE5Is (Ozdal et al., 2008; Kumar et al., 2015). It was reported by Ozdem et al. that combination therapy of PTX and sildenafil improved the management of vasculogenic ED of various types and degrees compared to sildenafil only (Ozdal et al., 2008). However, its study design lacks randomization, placebo, and PTX alone group, which are crucial in clinical studies. In another no placebo randomized trial, the authors indicated a much significant improvement in erectile function with combination therapy of tadalafil and PTX compared to tadalafil only in severe ED (Kumar et al., 2015). A randomized, double-blind placebo-controlled study showed that PTX had no augmentation role in ED patients who fail selective PDE5Is (Law et al., 2020). However, the trial did not achieve its desired sample size. Sildenafil is one of the main drugs used in ED treatment, while the non-respondence rate is not low and PTX is not effective as sildenafil in ED treatment (Drobnis and Nangia, 2017; Goldstein et al., 2019). PTX alone may be an efficient drug in the management of mild ED, and combined application of PTX and sildenafil may provide more erectile function improvement than monotherapy. In contrast, more studies were desired.

**TABLE 4:** The effect of oral PTX in ED.

Study	Study design	Type of ED	Intervention and control	Dosage (mg/d)	Duration of treatment (months)	No. of patients	Assessment for ED	Conclusion
Korenman and Viosca, (1993)	RCTs	Mild-to-moderate penile vascular insufficiency	PTX and placebo	3 × 400	3	24	Report of patient verified by partner as to number of coital episodes per month; penile-brachial pressure index determinations	PTX is a well-tolerated alternative therapy for erectile dysfunction in patients with mild-to-moderate penile vascular disease
Georgitis and Merenich, (1995)	RCTs	Diabetic ED	PTX and placebo	3 × 400	3	60	Serial self-appraisals of erectile function and objectively by nocturnal penile tumescence (NPT) monitoring	Pentoxifylline is not an effective treatment for diabetic erectile dysfunction
Peşkircioğlu et al. (1996)	RCTs	ED due to borderline arterial insufficiency	PTX and placebo	3 × 400 PTX	2	36	Penile duplex ultrasonography	Pentoxifylline was well tolerated. Oral PTX for 2 months could increase penile arterial inflow
Knoll et al. (1996)	RCTs	Mixed vasculogenic ED (arterial insufficiency and cavernous venous leakage)	PTX and yohimbine + isoxsuprine	3 × 400	2	20	Penile biothesiometry and penile duplex ultrasound scanning	Well-tolerated but no complete response to PTX. The overall partial response rate was 35% (7/20)
Ozdal et al. (2008)	Prospective study	Vasculogenic ED (various degrees and types)	PTX + sildenafil and sildenafil	3 × 400	1	68	International index of erectile function	Combination therapy of PTX and sildenafil improves the management of patients compared to sildenafil only
Zahran et al. (2012)	RCTs	After a T-shunt procedure for prolonged ischaemic priapism	PTX and placebo	3 × 400	3	40	Sexual Health Inventory for Men score	PTX had no significant effect on the recovery of EF because of too few patients and the nature of the disease (ischaemic injury rather than fibrosis only). Larger studies are required
Kumar et al. (2015)	RCTs	Severe ED	PTX + tadalafil and tadalafil	3 × 400	2	237	Self-administered IIEF-5 questionnaire	Much significant improvement in erectile function with combination therapy
Law et al. (2020)	RCTs	ED with suboptimal treatment response to sildenafil	PTX + sildenafil and sildenafil	3 × 400	2	50	IIEF-5 and the IIEF-15 score	Combination therapy of PTX and sildenafil does not improve the management of patients compared to monotherapy

Abbreviations: PTX: pentoxifylline; ED: erectile dysfunction; IIEF: International Index of Erectile Function.

### Adverse Events of Pentoxifylline

Most studies reported no AEs when using PTX for male infertility and ED. Four studies recorded detailed AEs with only one in male infertility treatment (shown in Table 5) (Peşkircioğlu et al., 1996; Safarinejad, 2011; Kumar et al., 2015; Law et al., 2020). Totally, PTX is a well-tolerated orally administered drug (100.00%), and its AEs, including nausea, vomiting, dyspepsia, headache, myalgia, diarrhea, tremor, dizziness, vertigo, backache, flushing, and leg pains, are mostly mild, transient, and minimal, which will not lead to drug discontinuity. Notably, PTX, a peripheral vasodilator, could induce hypotension. Thus, blood pressure should be monitored during treatment.

**TABLE 5:** Summary of AEs in the treatment of ED and male infertility with oral PTX.

Study	Adverse events
Peşkircioğlu et al. (1996)	Two patients had nausea and two others experienced headache
Kumar et al. (2015)	Few minor adverse effects were noticed in each group. No significant difference in between the two groups (tadalafil and combination of tadalafil and PTX) with regard to the occurrence of side effects. AEs included headache, back pain, nasal stuffiness
Law et al. (2020)	Gastrointestinal, neurological, musculoskeletal, and dermatological AEs were 4, 4, 2, 2, and 2, 1, 0, 0 in the combination group (sildenafil and PTX) and sildenafil group, respectively. No significant differences were observed between the two groups
Safarinejad (2011)	AEs included nausea, vomiting, dyspepsia, headache, diarrhea, tremor, dizziness, and vertigo. PTX was well-tolerated. The nature of adverse events was mild or moderate in severity. No serious adverse events were noted. None of the subjects discontinued the medication because of adverse effects

Abbreviations: PTX: pentoxifylline; ED: erectile dysfunction; AEs: adverse events.

## Discussion

In the study, we systematically reviewed the treatment role of PTX in male infertility and erectile dysfunction. Although meta-analysis was not conducted, we could still speculate the impact of PTX in the treatment of poor sperm quality and erectile dysfunction through narrative analysis; that is, PTX, with few mild AEs, is a well-tolerated drug in both ED and male infertility treatment. Furthermore, given the tight association between male infertility and ED, especially mild ED, we believed that PTX alone may improve sperm quality and erectile function in infertile men with ED, and drug combination application may achieve better treatment outcomes. Forrest plots with no synthesis results are shown in Supplementary Figures S1–S4 to clearly present the effect of PTX on erectile function improvement and sperm parameters among included studies. More well-designed studies with modern uniformed criteria using objective measurement, such as the computer-assisted sperm analysis (CASA), are warranted, and measuring ROS, antioxidants, and DNA integrity before, during, and after PTX administration may help understand the mechanism.

**FIGURE 3:**
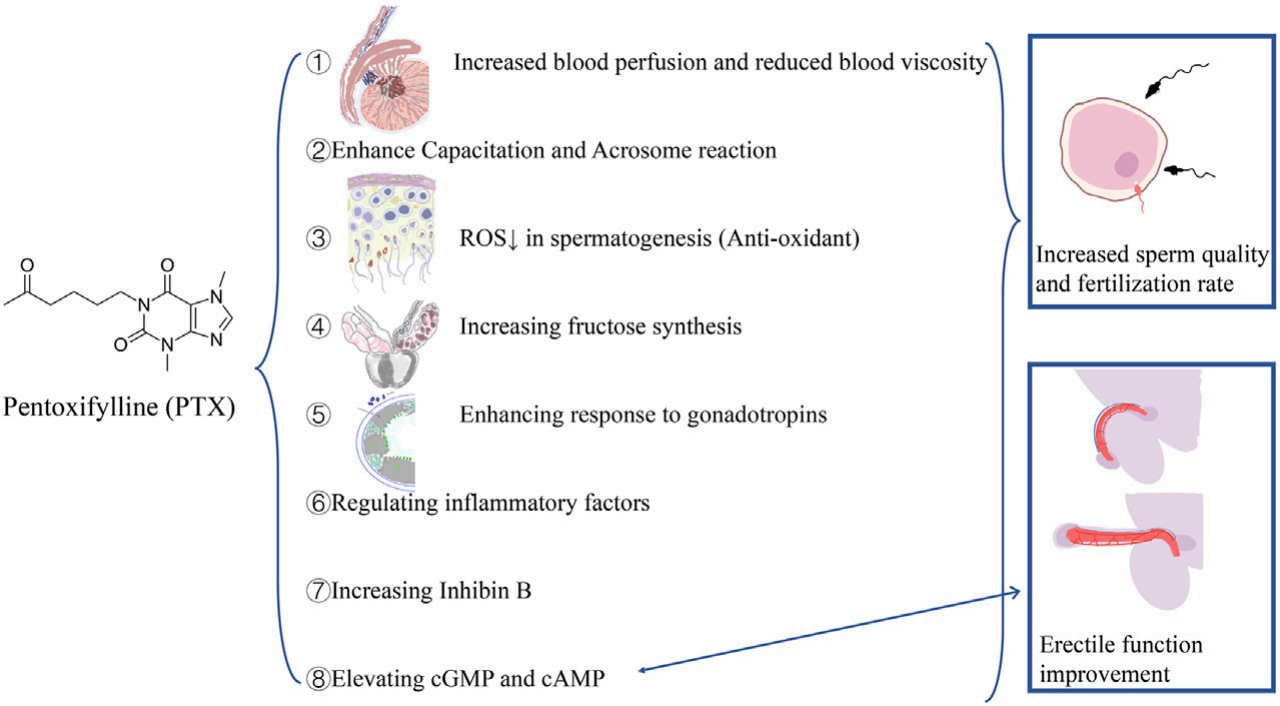
Mechanisms of oral PTX in the treatment of poor sperm quality and erectile dysfunction.

The effect of PTX on spermatogenesis and erection can be summarized as follows (shown in Figure 3): 1) inhibiting AMP catabolism and elevating cyclic AMP concentration in spermatogenic tissue (Aparicio et al., 1980a; De Turner et al., 1978): capacitation is a cAMP-dependent process; through this way, sperms acquire the fertilization ability (Lefièvre et al., 2002). 2) Direct effects on spermatogenetic tissues and cells: in 1980, Aparicio and his colleagues reported a direct metabolic change in sperms after the application of PTX *in vitro* (Aparicio et al., 1980b). It was reported that *in vitro* use of PTX can improve sperm motility and enhance acrosome reaction at a millimole concentration (Rees et al., 1990; Tesarik et al., 1992a; Tesarik et al., 1992b; Kay et al., 1993; Lewis et al., 1993; Moohan et al., 1993; Pang et al., 1993; Yovich, 1993; Tournaye et al., 1994). However, Schramm et al. reported that the maximum PTX concentration in seminal plasma was only 75 ng/ml (0.3 μM) (Schramm and Hinze, 1980). Recent two *in vitro* studies demonstrated the protective impact of PTX on spermatogenesis in mice models with both concentration levels higher than 0.3 μM (Dhulqarnain et al., 2021; Malekzadeh et al., 2021). 3) Increased blood perfusion and reduced blood viscosity in testes and epididymis: Pozor and his colleagues confirmed that PTX-treated stallions had significantly higher total arterial blood flow rate than controls, delaying the seasonal decrease of testicular perfusion, without sperm quality and total sperm volume improved (Pozor et al., 2011). 4) Anti-oxidation: reduced ROS levels are beneficial to spermatogenesis (Saleh and Agarwal, 2002). 5) Stimulating seminal vesicle epithelial tissues and increasing fructose synthesis (Marrama et al., 1985). 6) Leading to PDE metabolism inhibition in Leydig and Sertoli cells, enhancing response to gonadotropins, increasing local androgen and its binding proteins production, and maintaining spermatogenesis (Schill, 1979). 7) Regulating inflammatory factors, such as interleukins (ILs) and TNF-α. IL-6 and IL-8 play an important role in leukocytes recruitment and activation, and TNF-α can induce hydrogen peroxide produce from mitochondria (Comhaire et al., 1999; Jackson et al., 2002). High levels of ROS can be generated by activated leukocytes, overwhelming the anti-oxidation and leading to oxidative stress (Aitken et al., 1994). PTX can inhibit IL-6 and interfere with the synthesis of IL-8 (Crouch and Fletcher, 1992; D'Hellencourt et al., 1996; Sullivan et al., 1988; Thanhäuser et al., 1993; Zabel et al., 1989). What is more, PTX, as a xanthine derivative, can inhibit xanthine oxidase, which can facilitate the formation of the oxygen-free radical in sperm cells (Cross and Jones, 1991; Hammerman et al., 1999). 8) Increasing inhibin B, which is a marker of the seminiferous epithelium function and correlates with sperm concentration, indicating spermatogenesis improvement (Oates et al., 2002).

**FIGURE 4:**
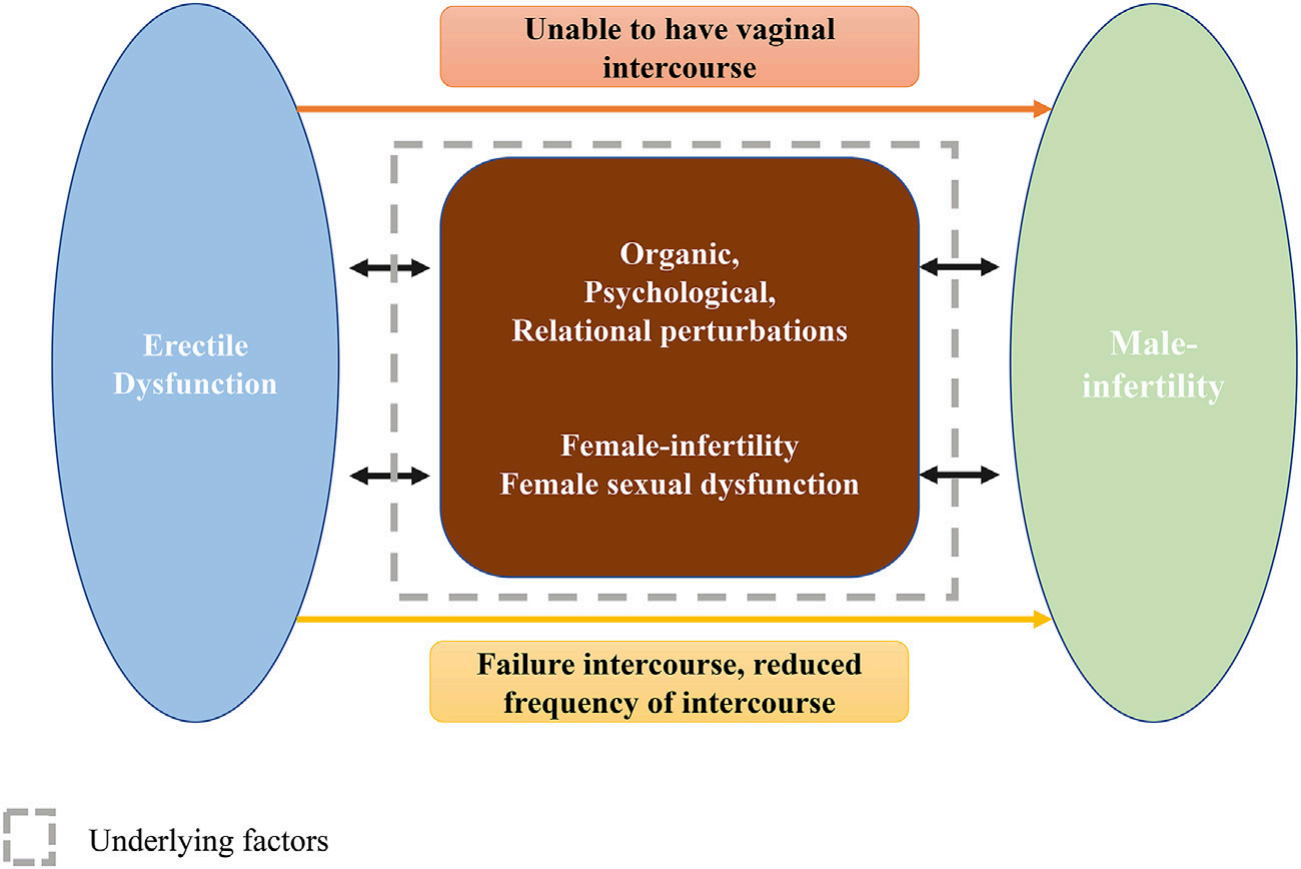
The interplay between ED and male infertility.; Abbreviations: ED: erectile dysfunction.

The association between ED and male infertility should be discussed (presented in Figure 4). Accumulating evidence has indicated the mutual cause-effect relationship between ED and male infertility, and studies have investigated the efficacy of oral PTX in male infertility with ED (Knoll et al., 1996; Safarinejad, 2011; Lotti et al., 2012; Zahran et al., 2012). ED is one of the causes of male infertility. When a severe ED is present, often manifested as insufficient erection for intercourse or even erection absence, ED leads to impaired fertility in natural conception. ED can be induced by organic (cardiovascular diseases, metabolic disorders, and neuroendocrine factors), psychological (anxiety or depression), and relational (in harmony within a family, etc.) perturbations (Yafi et al., 2016). Additionally, ED may lead to a dramatically reduced frequency of intercourse among infertile men, which definitely has a negative impact on fertility (Capogrosso et al., 2021). Furthermore, Li et al. found that the prevalence of ED among Chinese infertile men reached 57%, and most of them were mild ED. Furthermore, they indicated that infertile men often have more frequent intercourse during women’s ovulatory phase, while most of them reported failure of intercourse. They raised a novel concept, timely ovulatory intercourse failure (TOIF), and analyzed its prevalence and risk factors (Yang et al., 2018). They indicated that the TOIF prevalence was 26.2% in Chinese men of infertile couples and found that TOIF, correlating with lower sperm concentrations, might be a contributing factor of infertility (Yang et al., 2018; Xiong et al., 2020), which further illustrated the association between infertility and ED. Male infertility can also be a cause of erectile dysfunction. The prevalence of ED in male infertility has been investigated by several researchers. A previous review conducted by Lotti and Maggi (2018) summarized that the prevalence of ED in infertile men ranged from 11% (Saleh et al., 2003) to 69% (Berger, 1980a; Berger, 1980b) and that ED severity increased as semen quality impaired, being more severe in men with abnormal sperm parameters than in men with less abnormality, or with normozoospermia, or fertile men (Lotti et al., 2016; Niederberger, 2017). The large variation in prevalence is hard to determine and may be attributed to diverse evaluation tools, disparate sample sizes, and different races. Infertility can negatively affect sexual life, psychological status, and marital relationship of both partners (Lara et al., 2015; Berger et al., 2016), let alone a simultaneous sexual dysfunction occurred in female, which will further harm the couple’s sexual response. In fact, women among male infertility couples are more likely to experience decreased libido, shortness of breath, hypoxia, anxiety, depression, arousal, and orgasm dysfunction (Lara et al., 2015; Berger et al., 2016), which will conversely affect male erection ability.

The study may provide some clinical and research implications. 1) Male infertility is a common condition in men and is frequently accompanied by ED. The ED status and its accompanying psychological and organic disorders can also impair male fertility. In contrast, infertility status and its related psychological and relational concerns underlie ED. Previous evidence shows that modifiable causes of male infertility treatment can improve sperm quality and reverse infertility-associated ED. General health impairment can cause ED or male infertility, and they are both important proxies for general man health. ED in infertile men can be an early marker of poor health. Men with azoospermia have the highest psychological and general health disorders rates correlating with increased ED prevalence. General health, male fertility, and erectile function are closely associated. Studies focusing on psychological status, ED, and general health in men of infertile couples will improve reproductive, sexual, and general health. 2) PTX is a safe and tolerable drug. It is theoretically suitable for the treatment of male infertility with ED because of its pharmacological features, including anti-oxidation, anti-inflammation, increasing blood flow, AMP catabolism, and phosphodiesterase-5 inhibition. From the 1970s till now, there have been many investigations evaluating the role of PTX in ED or male infertility. However, the currently available evidence is insufficient and inconsistent to give a strong recommendation. Well-designed prospective RCTs investigating the role of PTX in infertile men with ED, dealing with fecundity, pregnancy rate, and erectile function improvement as major outcomes, are advisable. 3) ART brings not only opportunities but also challenges, especially to drug application in male infertility. ART lowered the fertility threshold. Thus, many infertile couples are offered ART without any evaluation or treatment of male infertility (Male Infertility Best Practice Policy Committee of the American Urological Association and Practice Committee of the American Society for Reproductive Medicine, 2006). Furthermore, because of limited knowledge about pathogenesis on infertility and conflicting findings from studies with various study designs, no consensuses were reached about oral drugs in male infertility treatment (Minhas et al., 2021). We should not stop our discovery in studying and proving the effectiveness of drugs. In turn, more high-quality evidence is expected to solve the dilemma.

## Conclusion

Mutually tight associations existed between male infertility and ED. They often occur in men simultaneously. In that case, control of the two diseases will bring great significance to patients and their families. PTX is a potentially effective and safe oral drug in the treatment of male infertility with ED. At the same time, it is currently an empirical treatment that achieves satisfying improvements in sperm quality in male infertility and erectile function in mild ED. PTX administration for the simultaneous treatment of poor sperm quality and mild ED in infertile men will highly enhance the treatment compliance. Combination therapies of PTX, selective PDE5Is, and other antioxidative drugs may further improve treatment outcomes.
