# Supplementary material for: Treatment of Poor Sperm Quality and Erectile Dysfunction With Oral Pentoxifylline: A Systematic Review
Source: Front Pharmacol. 2022 Jan 12;12:789787. doi: 10.3389/fphar.2021.789787 (PMC8790020; doi:10.3389/fphar.2021.789787)
Supplement: Supplementary file 1 [file DataSheet1.pdf]

Catalogue

Supplementary Table 1. Search strategy

Supplementary Table 2. RoB of cohort studies.

Supplementary Figure 1. Forrest plot of the effect of PTX on sperm concentration among included studies.

Supplementary Figure 2. Forrest plot of the effect of PTX on sperm motility among included studies.

Supplementary Figure 3. Forrest plot of the effect of PTX on sperm morphology among included studies.

Supplementary Figure 4. Forrest plot of the effect of PTX on ED among included studies.

Supplementary Table 1. Search strategy

	Search strategy
Population	#1 (((((((((Infertility, Male [MeSH Terms]) OR (Infertility, Male [Title/Abstract])) OR (Male Infertility [Title/Abstract])) OR (Sterility, Male [Title/Abstract])) OR (Male Sterility [Title/Abstract])) OR (Subfertility, Male [Title/Abstract])) OR (Male Subfertility [Title/Abstract])) OR (Sub-Fertility, Male [Title/Abstract])) OR (Male Sub-Fertility [Title/Abstract])) OR (Sub Fertility, Male [Title/Abstract])) OR (((((((((Erectile Dysfunction [MeSH Terms]) OR (Erectile Dysfunction [Title/Abstract])) OR (Dysfunction, Erectile [Title/Abstract])) OR (Male Sexual Impotence [Title/Abstract])) OR (Impotence, Male Sexual [Title/Abstract])) OR (Sexual Impotence, Male [Title/Abstract])) OR (Male Impotence [Title/Abstract])) OR (Impotence, Male [Title/Abstract])) OR (Impotence [Title/Abstract]))
Intervention	#2 (((((((((Pentoxifylline [MeSH Terms]) OR (Pentoxifylline [Transliterated Title])) OR (Oxpentifylline [Transliterated Title])) OR (Trental [Transliterated Title])) OR (Pentoxil [Transliterated Title])) OR (Torental [Transliterated Title])) OR (Agapurin [Transliterated Title])) OR (BL-191 [Transliterated Title])) OR (BL 191 [Transliterated Title])) OR (BL191 [Transliterated Title])
Comparator	Placebos or other drugs in addition to PTX

Outcomes	Measurement of sperm parameters, or erectile function, or hormone levels, or pregnancy outcomes
Study design	Randomized controlled trials, controlled clinical trials, prospective and retrospective cohort studies
Search combination	#1 AND #2

Abbreviations: PTX: pentoxifylline.

Supplementary Table 2. RoB of cohort studies.

Study (first autor,year)	Study design	Selection	Comparability	Outcomes	Total
Micic 1988	Prospective study	3	2	2	7
Ozdal 2008	Prospective study	4	2	3	9

Abbreviations: RoB: risk of bias.

Supplementary Figure 1. Forrest plot of the effect of PTX on sperm concentration among included studies.

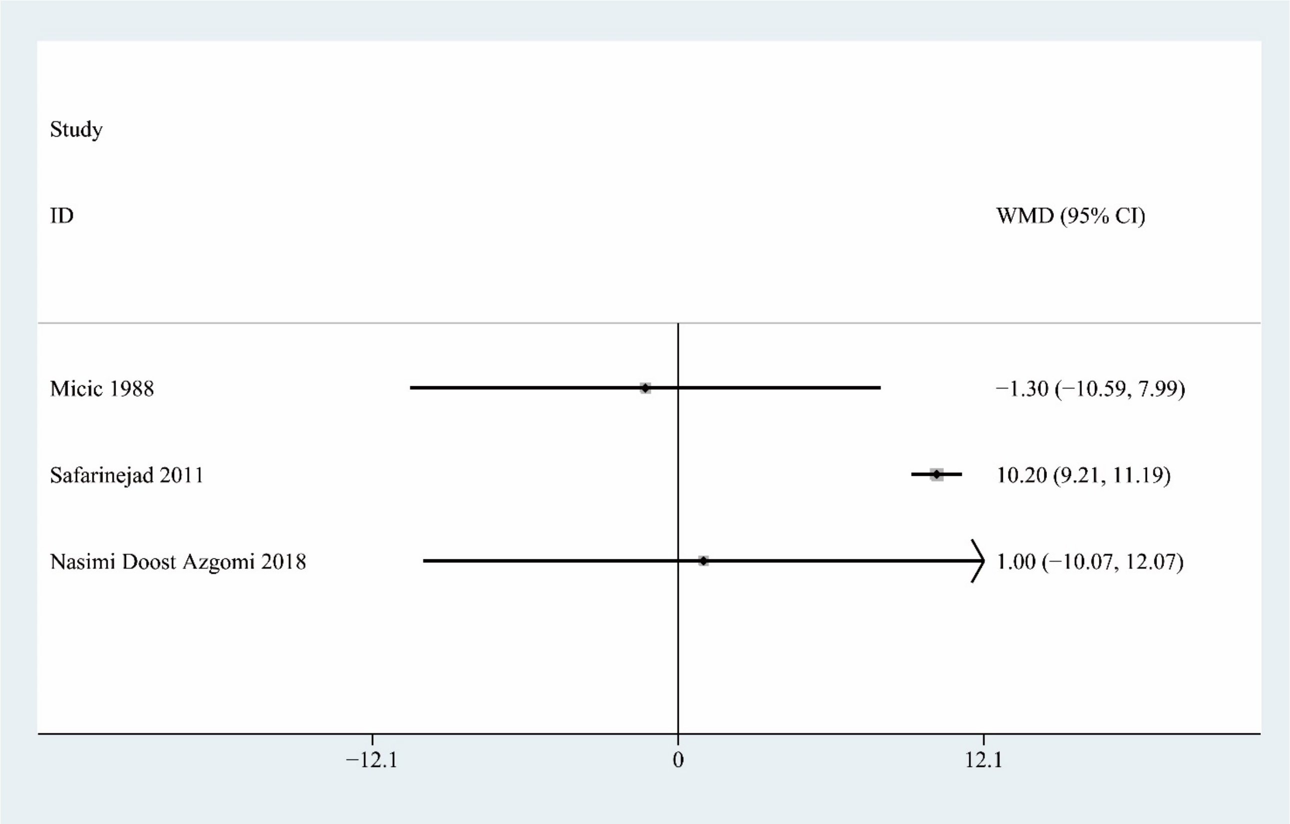

Supplementary Figure 2. Forrest plot of the effect of PTX on sperm motility among included studies.

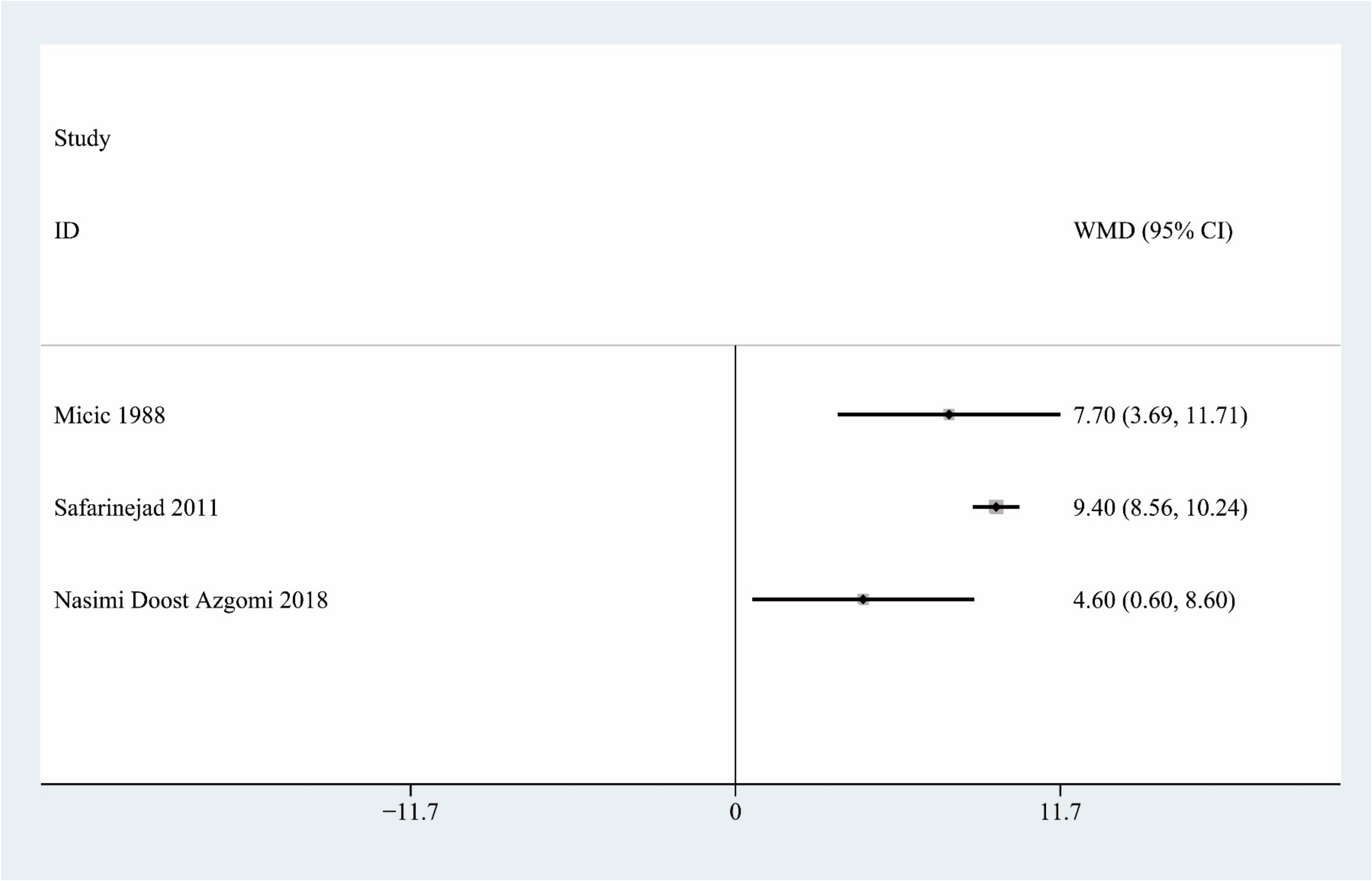

Supplementary Figure 3. Forrest plot of the effect of PTX on sperm morphology among included studies.

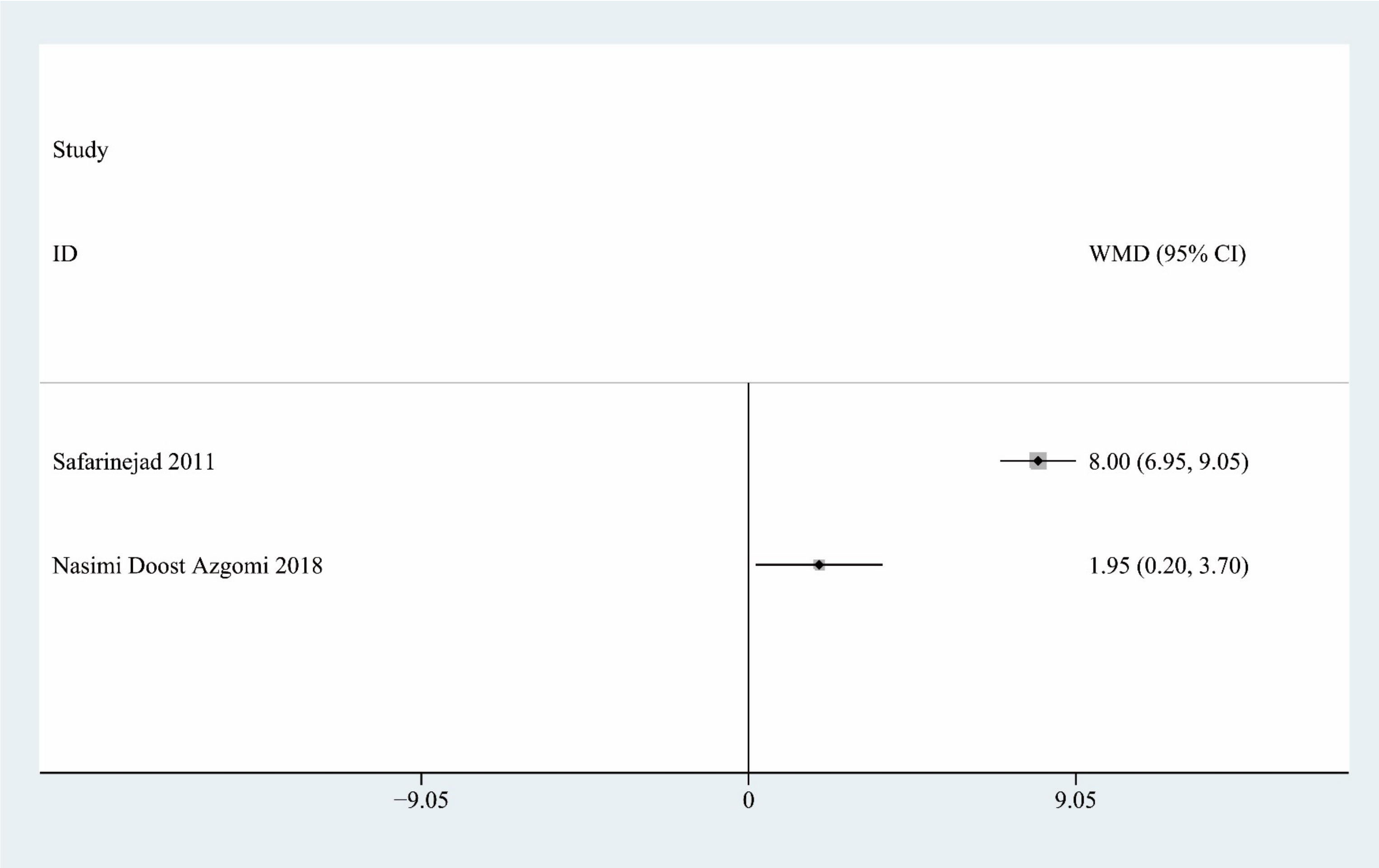

Supplementary Figure 4. Forrest plot of the effect of PTX on ED among included studies.

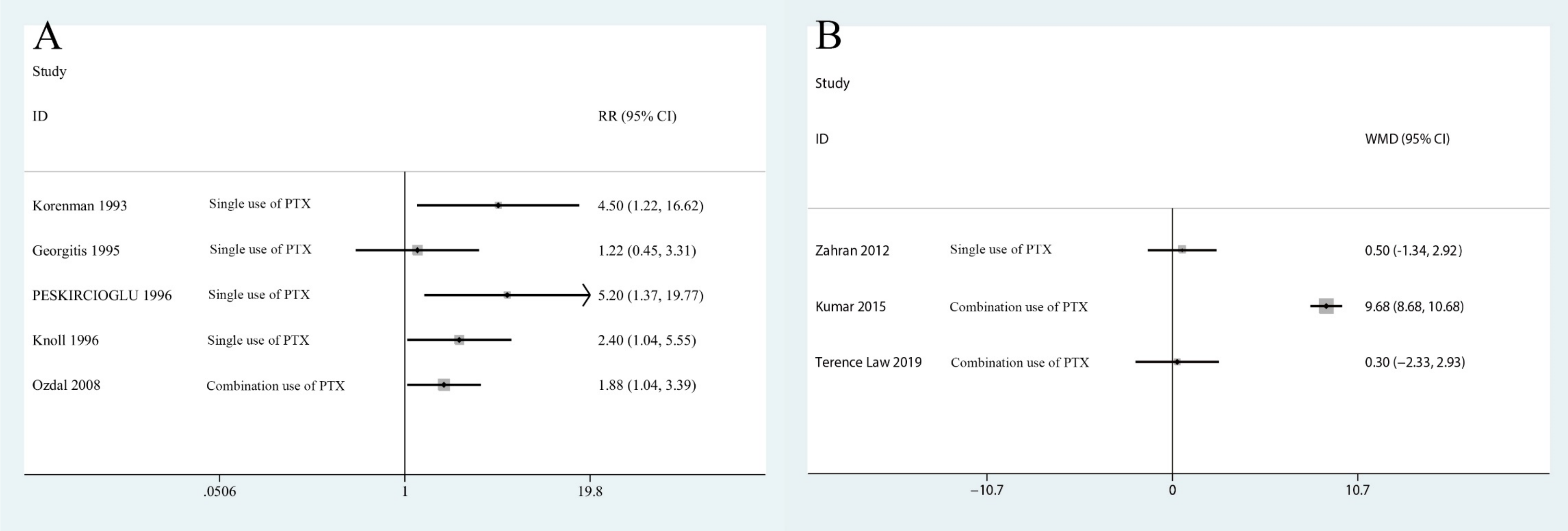

A: Studies reported erectile function outcomes in binary data
B: Studies reported erectile function outcomes through IIEF-5 scores.
